# Mind wandering during attention performance: Effects of ADHD-inattention symptomatology, negative mood, ruminative response style and working memory capacity

**DOI:** 10.1371/journal.pone.0181213

**Published:** 2017-07-24

**Authors:** Lisa M. Jonkman, C. Rob. Markus, Michael S. Franklin, Jens H. van Dalfsen

**Affiliations:** 1 Department of Cognitive Neuroscience, Faculty of Psychology and Neuroscience, Maastricht University, Maastricht, The Netherlands; 2 Department of Neuropsychology and Psychopharmacology, Faculty of Psychology and Neuroscience, Maastricht University, Maastricht, The Netherlands; 3 Department of Psychological and Brain Sciences, University of California, Santa Barbara, California, United States of America; Kings College London, UNITED KINGDOM

## Abstract

**Objective:**

In adulthood, depressive mood is often comorbid with ADHD, but its role in ADHD-inattentiveness and especially relations with mind wandering remains to be elucidated. This study investigated the effects of laboratory-induced dysphoric mood on task-unrelated mind wandering and its consequences on cognitive task performance in college students with high (n = 46) or low (n = 44) ADHD-Inattention symptomatology and Hyperactivity/Impulsivity symptoms in the normal range.

**Methods:**

These non-clinical high/low ADHD-Inattention symptom groups underwent negative or positive mood induction after which mind wandering frequency was measured in a sustained attention (SART), and a reading task. Effects of ruminative response style and working memory capacity on mind wandering frequency were also investigated.

**Results:**

Significantly higher frequencies of self -reported mind wandering in daily life, in the SART and reading task were reported in the ADHD-Inattention symptom group, with detrimental effects on text comprehension in the reading task. Induced dysphoric mood did specifically enhance the frequency of mind wandering in the ADHD-Inattention symptom group only during the SART, and was related to their higher self-reported intrusive ruminative response styles. Working memory capacity did not differ between high/low attention groups and did not influence any of the reported effects.

**Conclusions:**

These combined results suggest that in a non-clinical sample with high ADHD-inattention symptoms, dysphoric mood and a ruminative response style seem to be more important determinants of dysfunctional mind wandering than a failure in working memory capacity/executive control, and perhaps need other ways of remediation, like cognitive behavioral therapy or mindfulness training.

## 1. Introduction

Attention-Deficit/Hyperactivity Disorder (ADHD) is a persistent neurodevelopmental disorder characterized by impairing symptoms of inattention and hyperactivity-impulsivity, affecting 5% of children and adolescents and 2,5% of adults worldwide [1].

Also within non-diagnosed populations, such as college students, 2 to 8% reports clinically significant levels of ADHD symptoms associated with higher levels of functional, social and academic impairment [2]. Among ADHD patients, there however is large heterogeneity in symptom profiles, co-morbidity and underlying neuropsychological deficits. Inattention symptoms, as measured by DSM-IV based rating scales, seem to be more stable across development than hyperactivity-impulsivity symptoms [3], but also here identification of potential different neuropsychological attention subtypes is of crucial importance for the development of individually-tailored and targeted treatments.

As suggested by some authors, one such possible distinctive attention disorder underlying ADHD attention problems might be a pathological form of mind wandering [4]. Ceaseless and uncontrolled thought processes have been noted as an important characteristic in adult ADHD [5]. Mind wandering, or daydreaming, is a common everyday experience during which one’s attention becomes disengaged from the immediate external environment by unintentionally drifting away to stimulus-independent internal trains of thought (for recent review of the attention mind wandering literature see: [6]. Especially in tasks or situations that demand high levels of focused attention or cognitive control, such as during reading or in simple, non-engaging sustained attention tasks, undeliberate mind wandering deteriorates performance (for a review see [7]). Also in educational settings, such as when listening to a lecture, mind wandering has been shown to negatively affect memory and learning [8] [9] [10]. Since 2 to 8% of college students report clinically significant levels of ADHD symptoms associated with higher levels of functional, social and academic impairment [2], it is important to investigate pathological mind wandering as a possible underlying cause for their attention problems.

To the best of our knowledge, only four studies have yet investigated links between mind-wandering and ADHD symptoms, three in populations of college students ([11–13] and one including ADHD patients [14]. In a first study, Shaw and Giambra [11] investigated mind wandering during cognitive performance among three groups of undiagnosed college students with either high or low hyperactivity symptoms or with a self-reported childhood ADHD diagnosis. Mind wandering (spontaneous/deliberate) was measured by presenting thought probes during a sustained attention task asking participants to indicate whether they had Task Unrelated Thoughts (TUT’s) or not. Students with childhood ADHD reported the highest number of spontaneous (but not deliberate) TUT’s and made more false alarms. Furthermore, those with high, compared to low, self-reported hyperactivity symptoms also reported more spontaneous TUT’s. In a recent association study [12], ADHD-symptom and mind wandering data were collected from a random sample of 105 college students. A composite ADHD-symptom measure was computed on the basis of two self-report adult ADHD questionnaires and level of mind wandering was measured by 1) self-report trait questionnaires 2) thought probes presented during a sustained attention and reading task and 3) thought probes presented 8 times a day for a week on a digital device carried by the participants. ADHD symptoms correlated highly positive (.68) with a composite trait mind wandering score based on questionnaires and moderately positive (.24) with a composite lab/daily life state mind wandering score. In another association study [13], two samples of 1354 college students each filled in a short 6-item screener to measure ADHD-symptoms and two questionnaires measuring trait levels of deliberate and spontaneous mind wandering. Only spontaneous mind wandering showed a positive correlation with ADHD-symptoms In a fourth questionnaire study [14], significantly higher scores on a newly developed questionnaire measuring a pathological, excessive form of mind wandering were reported by diagnosed ADHD patients than controls. Hierarchical regression analyses across ADHD and control groups showed that only ADHD-Inattention symptoms and Mind Wandering scores explained unique variance in reported functional impairment. Concluding, the above discussed studies provide consistent evidence for associations between ADHD symptomatology and spontaneous undeliberate mind wandering in daily life and during cognitive performance, but also leave some unexplored issues.

First, prior studies were cross-sectional in nature and did not use an experimental design allowing for the study of directional relations between study variables. Second, by only focusing on hyperactivity or combined-ADHD symptoms, none of the above studies investigated the specific role of ADHD-inattention symptoms in reported ADHD–mind wandering relations and its detrimental effects on cognitive performance. The latter is important because of the potential role of co-occurring internalizing psychopathology such as depression that is more strongly associated with inattentive than hyperactivity/impulsivity ADHD symptoms [3, 15]. Depressive symptoms have been shown to enhance mind wandering during attention, memory encoding or silent reading tasks [16–18] and laboratory induced sadness has been shown to lead to exacerbated task-irrelevant thoughts with detrimental effects on cognitive performance in healthy, non-depressed, young adults [19–21]. Previous ADHD–mind wandering studies did not take such effects of mood or individual differences in depressive symptoms into account. The present study will fill this gap by experimentally investigating effects of ADHD-Inattention symptoms and negative mood on mind wandering frequency during cognitive task performance while taking individual differences in depression into account. For this purpose, two groups of college students with extreme scores on the ADHD-Inattention symptom scale, but normal scores on the hyperactivity-impulsivity symptom scale, will be a priori selected for participation in the lab experiment.

Potential detrimental effects of negative mood on mind wandering and cognitive performance might be even stronger in individuals that use maladaptive strategies to deal with negative emotions or events, such as when having an intrusive/ruminative response style. Individuals with high dysphoric mood or a tendency towards depression are often characterized by automatic, non-adaptive (intrusive and repetitive) ruminative thinking styles, also referred to as brooding [22]. In the mind wandering literature, Smallwood & Andrews-Hanna [23] proposed the *content-regulation* hypothesis, suggesting that individual differences in information processing styles might influence mind wandering content; those with non-adaptive processing styles being more prone to ruminating about negative events. There is some evidence from non-clinical samples for lower trait levels of mindfulness or higher levels of mind wandering being related to non-adaptive/negative thinking styles. In one study [24], those reporting lower levels of mindfulness also reported a higher frequency of automatic, hard to suppress, negative thoughts. In another recent study self-reported daydreaming frequency was uniquely related to brooding and not to adaptive, reflective thinking styles [25]. To our knowledge, the role of negative mood and intrusive ruminative thinking styles in ADHD-inattention—mind wandering—cognition relations has not yet been examined.

A third factor of potential importance that is not taken into account in above reviewed studies is the ability to exert top down control over mind wandering which is determined by one’s executive or working memory capacity. In general it is found that adults with higher working memory capacity experience less episodes of task-unrelated mind wandering during complex cognitive tasks like reading, or tasks requiring focused attention for longer periods of time such as the sustained-attention to response task (SART) [26–29]. Working memory capacity deficits have been reported in adult ADHD (see meta-analysis [30]), and multiple studies report specific links between executive working memory deficits and inattention symptoms/inattentive behavior in children or adults with ADHD ([31–35]. Considering the above, individual differences in working memory capacity might play an important role in putative ADHD-inattention, mood, mind wandering relations and hence will also be investigated in the present study.

Summarizing, the present study investigated differences in mind wandering in relation to negative mood and ruminative thinking between groups of non-diagnosed young adults with high or low ADHD-Inattention symptoms but ADHD-Hyperactivity/Impulsivity scores in the normal range and matched/controlled for between groups. Half of each attention group was exposed to either negative or positive mood induction followed by performance of a sustained attention to response task (SART) and a reading task while measuring mind wandering (TUT’s) via thought-probes and an often used post-task questionnaire. In order to replicate earlier reports of negative mood induction in the SART especially enhancing TUT’s about events in the past, as opposed to those in the present or future, we included the same temporally oriented (present, past, future) thought probes in the SART. In the reading task we included regular on and off-task thought probes. Trait levels of mind wandering and ruminative thinking styles were assessed by questionnaires and working memory capacity was measured by the Operation Span task [36]. The ADHD-Inattention symptom group is hypothesized to show higher trait and state mind wandering (TUT’s) than the low symptom group, higher state mind wandering leading to worse cognitive performance. Negative mood and a ruminative thinking style are hypothesized to strengthen such effects in those with high ADHD-Inattention symptoms. Since in the developmental ADHD-literature links between attention control problems and (a lack of) working capacity have been reported, potential working memory capacity differences between the groups will also be measured and taken into account in the analyses.

## 2. Materials and methods

### 2.1. Participants

A group of 850 university students completed an online questionnaire package, including two self-report ADHD-symptom lists; the ACTeRS and a Dutch-normed DSM-IV based rating scale (and also other questionnaires mentioned below). Potential participants for the present experimental study were selected from the database on the basis of their score on one of the ADHD-self report questionnaires (the ACTeRS). To form two attention groups with either high or low ADHD-inattention symptoms, participants were invited for participation in the study if they had raw scores falling in either the lowest or the normal/highest population percentiles on the ACTeRS Attention subscale. Based on these criteria two attention groups were formed; a group represented by high ADHD-Inattention symptoms (hereafter called the *ADHD-IA symptom group* consisting of n = 46 (11m/35f), with ACTeRS-Attention raw scores ranging between 19–29, M = 25,8 (SD = 2,8); these scores fall in the 10^th^ and 20^th^ population percentile (lower scores indicating more problems). In the second group participants had no ADHD-inattention symptoms (*low symptom group* consisting of n = 44 (7m/37f), with ACTeRS-Attention raw scores ranging between 38–48, M = 41,0 (SD = 2,1), all scores falling in the 80^th^ and 90^th^ population percentiles. Because, as explained in the introduction, we were especially interested in ADHD-inattention–mind wandering links, all participants were a priori also required to have ACTeRS Hyperactivity/Impulsivity (H/I) subscale scores above the problem range, according to the ACTeRS manual defined as scores falling in or above the 35^th^ percentile, which would correspond with a raw score of 43 or higher; for mean raw scores (and SD’s) in both groups see Table 1. Thirteen participants in the *ADHD-IA symptom group* and four in the *low symptom group* did not reach the criterion of H/I raw score ≥ 43). Because exclusion of these 17 participants did not change below reported ANOVA results for the SART or reading tasks in important ways, all participants were included in the analyses to have maximum power and equally sized groups; note that mean raw H/I scores in both groups were in the normal range and on a second ADHD scale no difference in Hyperactivity/Impulsivity scores was found (see Table 1). To further validate the ACTeRS attention group formation, a second DSM-IV based adult ADHD self-report rating scale was administered (see below for description); confirming significant group differences in only Inattention scores and not Hyperactivity/Impulsivity scores (see Table 1). This study has been performed according to the principles expressed in the Declaration of Helsinki and written informed consent was obtained from all participants (all were above 18 years of age). All participants were informed about the study by an invitation mail, on the basis of which they could enroll themselves in the study. Upon arrival in the lab they again received information about the study procedure after which they signed the informed consent form. The present study, including the above consent procedure, was approved by the Ethical Review Committee Psychology and Neuroscience (ERCPN), Maastricht University, The Netherlands (approval number ECP-125_14_02_2013).

**Table 1:** Scores and differences between the two attention groups on the different questionnaires filled in before the lab visit and on the OSPAN working memory test performed during the lab session (see text for explanation of abbreviations and content of the questionnaires). IA = Inattention, H/I = Hyperactivity/Impulsivity.

	ADHD-IA high symptom group (N = 46)		ADHD-IA low symptom group (N = 44)		Attention Group effects (indep. t-test stats)		
	Mean	SD	Mean	SD	t	*p-value*	*ES (Cohen’s* d)
Age (months)							
ADHD_IA (Acters*)	25,8	2,8	41,0	2,1	-29,2	< .0001	6.1
ADHD_H/I (Acters)	46,3	6,2	49,4	5,6	-2,4	.02	0.5
ADHD_IA (ZRV*)	10,2	4,1	4,2	3,1	7,7	< .0001	-1.6
ADHD_H/I (ZRV)	10,2	4,5	9,1	4,1	1,2	.21	-0.3
ARCES_mean score	2,7	0,5	2,2	0,4	5,0	< .0001	-1.1
MAAS_ mean score	3,7	0,6	4,4	0,7	-5,6	< .0001	1.1
DDFS_mean score	1,8	0,7	1,2	0,5	4,9	< .0001	-1.0
BDI- total depression score	8,1	6,7	4,4	4,0	3,2	.002	-0.7
ERRI_intrusive_total score	12,1	7,8	7,7	4,5	3,2	.002	-0.7
ERRI_deliberate_total score	12,1	6,4	10,9	5,8	0,9	.37	-0.2
OSPAN (working memory)**	42,4	10,9	42,8	11,6	-0,1	.84	0.0

*Raw scores for both scales are presented. Note that on the ACTeRS-Inattention subscale (on the basis of which the present attention subgroups were initially formed) a lower score indicates more attention problems whereas this is reversed on the ADHD_ZRV (that was included as an additional ADHD questionnaire).

** Presenting absolute OSPAN scores. Two participants in the ADHD-IA high symptom group and one in the low symptom group had zero scores in the OSPAN so these scores (and t-test) are based on groups of N = 44 and N = 43 respectively.

### 2.2. Procedure

All participants fulfilling the inclusion criteria as mentioned above (see participants section) were informed about the study and invited for participation via e-mail and could enroll themselves for the study via an electronic agenda. When arriving at the lab all participants were first informed about all procedures and all provided written informed consent for participation. Half of the participants in each attention group were randomly assigned to either the positive or negative mood induction condition. The session started with the OSPAN task, after which the participants were exposed to positive or negative mood induction, followed by the sustained attention task (SART) and reading task, while assessing mind wandering (TUT’s) via online thought probes and a post-task questionnaire (DSSQ; see paragraph 2.3.2.3). After the SART, before the reading task, the mood induction procedure was repeated with the same emotional valence and sentences as one had before, but with different music pieces. Mood state changes were measured by the PANAS at four different times during the experiment: directly before and after the first mood induction and following the SART and the reading task. The DSSQ was filled in directly after the SART and reading task, always before the mood questionnaires (PANAS and POMS). After completion of the session, participants in the negative mood condition underwent positive mood induction for recovery.

### 2.3. Materials and tests

#### 2.3.1. Questionnaires

The following questionnaires were completed online by the sample of 850 college students, several months before the experimental session of the present study was conducted.

**2.3.1.1. ADHD questionnaires.** The Attention and Hyperactivity/Impulsivity subscales from the ACTeRS, a DSM-IV based ADHD self-report questionnaire (ACTeRS; Metritech, Inc), was used for a priori selection of study participants. This instrument contains three subscales measuring: Attention problems (10 items), Hyperactivity/Impulsivity (15 items) and Social Adjustment (10 items) using a five point scale varying from Strongly Disagree (1) to Strongly Agree (5).

A second DSM-IV based ADHD- self report rating scale (ZRV) developed and validated in a Dutch population sample [37], was used to confirm the validity of our attention group formation.

**2.3.1.2. Trait mind wandering/daydreaming/mindfulness questionnaires.** The 12-item Attention Related Cognitive Errors Scale (ARCES; for further description see [38] measures the frequency of attention related errors in daily life due to mind wandering. The 15-item Mindful Attention Awareness Scale (MAAS; for further info see [39] and the 12-item Day Dreaming Frequency Scale (DDFS; see [40] were used to respectively measure trait mindfulness and daydreaming frequency.

**2.3.1.3. Depression and rumination questionnaires.** The Beck Depression Inventory (BDI-1; for details see [41]) measured depression symptoms experienced in the last week. The 20-item Event Related Rumination Inventory (ERRI; for details see [42]) contains two subscales measuring the frequency of 1) adaptive deliberate/reflective ruminative thoughts and 2) non-adaptive intrusive/brooding ruminative thoughts during negative life events, on a 4 point scale ranging from 0 (not at all) to 3 (often).

#### 2.3.2. Laboratory tests and manipulations

**2.3.2.1. Working Memory task.** The Automated Operation Span (OSPAN) test developed by Unsworth et al., [36] was used as a working memory measure. In the current analyses the absolute OSPAN score, the sum of completely correctly recalled sets, was used as Working Memory Capacity measure, see for norm scores [43].

**2.3.2.2. Mood induction.** The mood induction procedure from Mayer et al. [44] was used. Participant’s listened to either sad or happy music through headphones while reading self-referent sad or happy statements that were occasionally presented at the screen. Music pieces for the negative condition were either Samuel Barbers’ *Adagio for Strings* or Sergej Prokofiev’s *Russia under the Mongolian Yoke* (the latter played half speed). Music pieces for the positive condition were *Eine Kleine Nachtmusik* by Mozart or *The Four Seasons* by Vivaldi [45]. For each condition, eight different self-referent statements were used from [46]. Examples of positive and negative statements are respectively: ‘You buy a lottery ticket and you win a $100 instantly’ and ‘A pet you were really fond of has died’. The present mood induction procedure has been successfully used in a prior mind wandering study [21].

The Positive Affect Negative Affect Schedule (PANAS; [47]) and the shortened Profile of Mood States (POMS) questionnaires were used to monitor mood state changes at multiple times during the experiment (see paragraph 2.2). Since the PANAS and POMS yielded similar results, for space reasons the POMS data will not be further reported.

**2.3.2.3. Sustained Attention to Response task (SART)**. The SART was originally developed by Robertson et al. [48]. In compliance with the literature, a computerized SART task with online thought probes was used to measure mind wandering frequency during sustained attention (see [49]). A total of 615 digits were presented in the centre of a computer screen of which 549 were targets (e.g. digits 1, 2, 4, 5, 6, 7, 8 or 9) to which one had to respond by pressing the space bar, and 66 were non-targets (the digit 3: frequency of 10%) to which one had to refrain from responding. All digits were presented in random font sizes. Stimulus presentation time was 500 milliseconds and the response window varied between 900 and 1200 milliseconds. Fifteen thought probes were randomly inserted in between trials to measure temporal orientation of mind wandering (e.g.: ‘*At this moment*, *what are your thoughts related to*?’) and participants had to press keyboard buttons 2, 4, or 6 to relate their thoughts respectively to the future, the present or the past.

In addition to the thought probes, the 16-item Thinking Content part of the Dundee Stress State Questionnaire (DSSQ; for details see [50]) was administered after the SART to obtain a second, retrospective measure of mind wandering. The DSSQ is a frequently used self-report measure of mind wandering and distinguishes between the frequency of Task Related Interfering Thoughts (TRI) or Task Unrelated Thoughts (TUT) during a previously performed task. Both types of thought are detrimental for task focus/performance, but only TUT’s are considered to be instances of (task-unrelated) mind wandering in the mind wandering literature [6]. Because of this important distinction a factor Thought Content will be included in the analyses of the DSSQ results. An example of a TRI item is: “I thought about the purpose of this task” and a typical TUT item is: “I thought about something that may happen in the future”. All responses were measured on a 5-point Likert scale ranging from “never” to “very often”.

**2.3.2.4. Reading task.** The second experimental task to measure participant’s mind wandering frequency was a translated and slightly shortened version (to 4342 words) of the task earlier used by Smallwood et al. [51]. The task consisted of a Dutch version of the Sherlock Holmes story ‘The Red Headed League’ that was displayed word by word on a computer screen. The participants could advance the text by pressing the space bar. During reading, 25 thought probes were presented after a randomly determined number of 70 to 250 words. The thought probes screens presented the question: “In the moments preceding this question did you have thoughts that were not related to the task (did your thoughts wander off)? “to which the participants responded by pressing 1 (yes) or 2 (no) buttons on the keyboard. The reading task was followed by 20 (of the original 23; see [51]) text comprehension questions in multiple choice format with four answer alternatives per question. Three questions were omitted because they referred to text deleted due to shortening. The complete reading task lasted about 30–45 minutes depending on an individuals’ reading speed. The task was programmed in e-prime 2.0. After the reading task the DSSQ was again administered to obtain a second, retrospective, measure of mind wandering during the task (for explanation of DDSQ see paragraph 2.3.2.3).

## 3. Results

### 3.1. Group differences on the trait questionnaires and OSPAN

Mean scores (and SD) in the different attention groups and results of the independent t-tests (and effects sizes) performed to test for group differences are presented in Table 1. The groups showed large differences on the Inattention subscales of both ADHD-rating scales, whereas scores on the Hyperactivity/Impulsivity subscale were either not significant (ADHD_ZRV) or weak and very low in effect size (ACTeRS). Furthermore, as predicted, the ADHD-IA symptom group reported significantly higher frequency of attention errors in daily life due to mind wandering (ARCES) and daydreaming (DDFS) and lower levels of daily mindfulness (MAAS) than the low symptom group. The ADHD-IA symptom group also showed higher levels of non-adaptive, intrusive ruminative response styles (ERRI-Intrusive) and depression (BDI) than the low symptom group, whereas adaptive, deliberate rumination scores did not differ between the groups. The groups also did not differ in working memory capacity (OSPAN) and all OSPAN scores fell within the normal (50^th^ percentile) population range; see [43].

### 3.2. Effects of mood induction on mood state

Analysis of variance (ANOVA) on PANAS mood scores before and after the first mood induction showed a three-way interaction effect of Mood induction (positive vs negative) x Time (pre vs post) x PANAS (positive vs negative scale) (F(1,83) = 5.3, *p* < .001, η_p_^2^ = .38). Follow-up tests for the separate mood induction conditions revealed a Time x PANAS interaction after Negative mood induction (F(1,43) = 58.6, *p* < .001, η_p_^2^ = .58); indicating a large decline in positive mood [from *M* = 26,5 (SD = 6,9) to *M* = 18,9 (*SD* = 8,3): t(43) = 8.4, *p* < .001] and an increase in negative mood [from *M* = 12.4 (*SD* = 2.5) to *M* = 14.8 (*SD* = 4.6): t(44) = -3.6, *p* < .002]. For positive mood induction, there also was a Time x PANAS interaction (F(1,42) = 4.6, *p* = .04, η_p_^2^ = .10); indicating a reduction in negative mood [from *M* = 12.1 (*SD* = 2.5) to *M* = 10.6 (*SD* = 2.2): (t(44) = 4.4, *p* < .001)] but no change in positive mood (*M* = 24.9 (*SD* = 7.1) and *M* = 25.8 (*SD* = 7.8); t(42) = -0.8, *p* = .42). There were no main or interaction effects including the Attention Group factor.

We also measured mood after the SART (T3) and reading task (T4) to monitor stability of mood induction effects. A first analysis compared mood at T2 (after the first mood induction, before the SART) with mood at T3 (after the SART, before the second mood induction) and yielded a Mood Induction x Time x PANAS interaction (F(1,82) = 63.5, *p* < .001, η_p_^2^ = .44); the negative mood induction group showed stability (no change) of negative mood from T2 to T3 (Time: *p* = .89 and Time x PANAS: *p* = .15), whereas the positive mood induction group showed reduced positive and increased negative mood (Time x PANAS: F(1,41) = 99.0, *p* < .001, η_p_^2^ = .44). A second analyses compared mood at T3 to that at T4 (after the reading task), positive mood did not change (Time: *p* = .72, Mood Induction x Time: *p* = .72) whereas negative mood declined in both groups (mean change of 2.4 points; Time: *p* < .001: Time x Mood Induction: n.s).

### 3.3. SART

#### 3.3.1. Effects on mind wandering measured by online thought probes

See Table 2 for Means (and SDs) for all dependent variables in the SART in the separate attention groups and mood induction conditions. A 2 (Attention Group) x 2 (Mood Induction) x 3 (Temporal Orientation: future, present, past) ANOVA analysis on *thought probe* scores during SART performance only yielded a main effect of Temporal Orientation (F(2,85) = 51.6, *p* < .001, η_p_^2^ = .55); indicating more thoughts about the present than future (t(89) = -7.1, *p* < .001) or past (t(89) = 10.2, *p* < .001) (the latter also being less frequent than future thoughts (t(89) = 2.6, *p* = .01)). There were no further main or interaction effects (all *p’s* >.12).

**Table 2:** Mean scores (and SD’s between brackets) for all dependent variables in the SART and reading tasks in the two attention groups and positive and negative mood induction (MI) conditions.

	ADHD-IA high symptom group		ADHD-IA low symptom group	
	positive-MI (n = 23)	negative-MI (n = 23)	positive-MI (n = 22)	negative-MI (n = 22)
*SART*				
go trials—% correct	98 (0)	99 (0)*	99 (0)	99 (0)
go trials-mean RT	330 (53)	331 (68)	326 (66)	340 (88)
Nogo- % CE	48 (2)	50 (2)	50 (2)	46 (2)
TP_present**	8,0 (3,6)	9,4 (3,0)	8,7 (4,4)	9,0 (4,8)
TP_future	3,9 (3,1)	3,4 (2,5)	3,9 (3,0)	3,7 (4,3)
TP_past	3,0 (2,3)	2,2 (1,8)	2,5 (2,9)	2,5 (3,0)
DSSQ_TRI	23,3 (4,5)	21,2 (4,6)	22,0 (4,9)	23,6 (4,6)
DSSQ_TUT	13,8 (4,2)	16,7 (5,5)	13,0 (4,3)	13,6 (3,3)
*Reading task*				
TP_1st half task***	4,1 (3,1)	4,9 (3,9)	2,5 (3,0)	3,3 (3,3)
TP_2nd half task	5,1 (4,3)	5,2 (4,3)	4,3 (3,1)	5,5 (4,3)
DSSQ_TRI	17,9 (5,2)	17,6 (5,7)	15,8 (4,6)	17,1 (4,5)
DSSQ_TUT	11,8 (3,7)	12,7 (4,1)	9,7 (2,3)	10,4 (4,1)
Comprehension-ACC	11,4 (3,0)	10,5 (4,1)	11,6 (2,5)	13,2 (2,9)

Abbreviations: IA = inattention; MI = mood induction; RT = reaction time; CE = commission errors; TP = thought probes; TRI = task-related interfering thoughts; TUT = task-unrelated thoughts; ACC = accuracy.

*SART performance data from one participant was disregarded due to use of wrong response buttons

**the data depicted is the number of yes responses given in each temporal category to the 15 probes.

***the data depicted is the mean number of thought probes on which mind wandering was reported.

#### 3.3.2. Effects on mind wandering measured by retrospective report (DSSQ)

**Fig 1:**
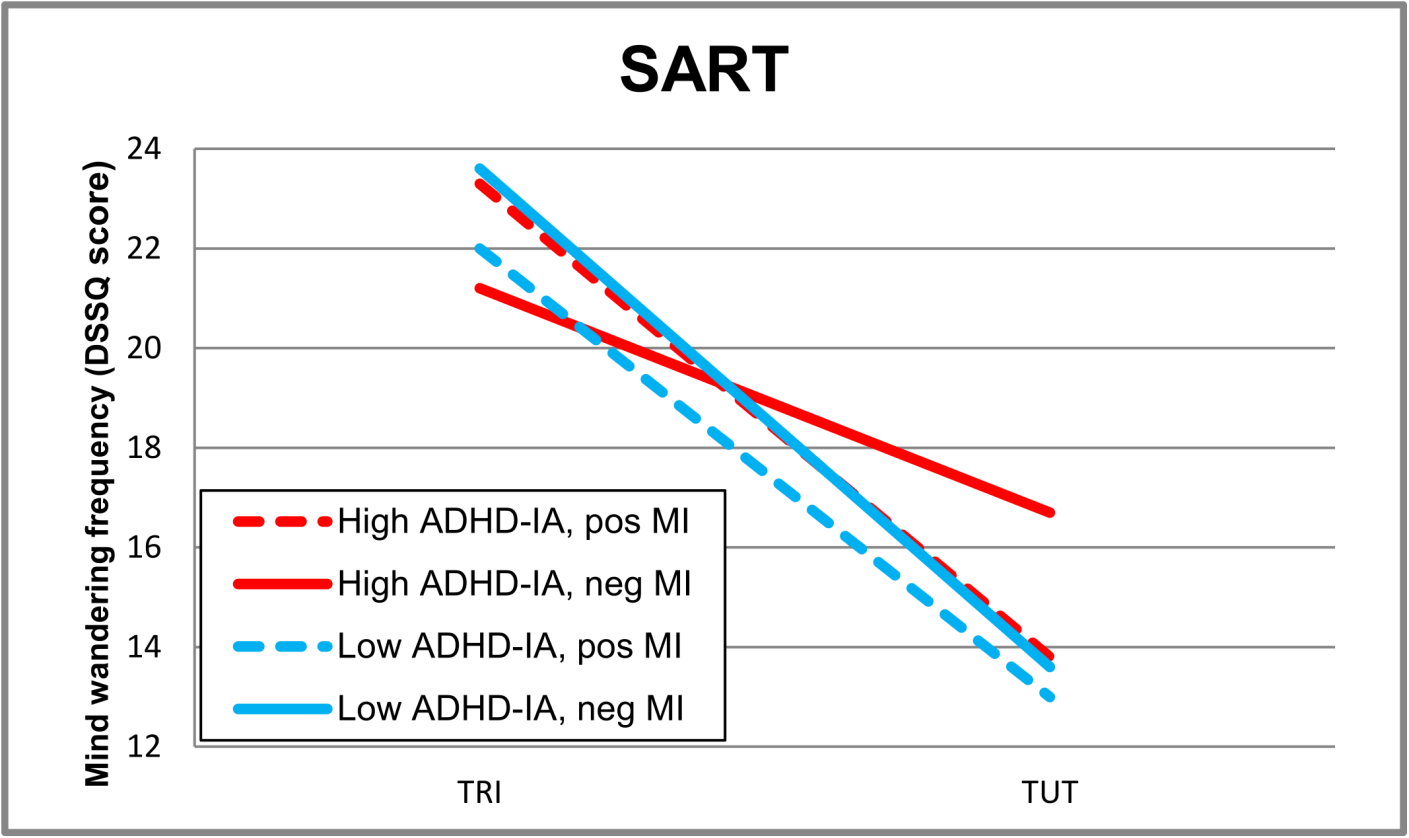
Mean Task-Related Interfering (TRI) and Task-Unrelated-Thought (TUT) scores on the post-task questionnaire (DSSQ) in the Sustained Attention to Response Task (SART) in high and low ADHD-Inattention (IA) symptom groups after positive (pos) or negative (neg) mood induction (MI).

To measure effects on *retrospective reports of TRI’s and TUT’s* during the SART (by DSSQ), a 2 (Attention Group) x 2 (Mood Induction) x 2 (Thought Content: TRI vs TUT) ANOVA was performed. Analysis revealed a main effect of Thought Content (F(1,86) = 196.0, *p* < .001, η_p_^2^ = .69), an interaction effect of Attention Group x Thought Content (F(1,86) = 4.5, *p* = .04, η_p_^2^ = .05) and a three-way interaction effect of Attention Group x Mood induction x Thought Content (F(1,86) = 6.3, *p* = .014, η_p_^2^ = .07: see Fig 1). The 3-way interaction was further explored by testing Mood Induction x Thought Content interactions in the separate attention groups, yielding a significant effect in only the ADHD-IA symptom group (F(1,44) = 8.6, *p* = .005, η_p_^2^ = .16). Further testing indicated higher TUT’s after negative than positive mood induction (F(1,44) = 2.3, *p* = .051, η_p_^2^ = .08) while TRI was not influenced by mood induction (*p* = .14). In the low symptom group, mood induction had no effects (*p* = .56). Inclusion of BDI or ACTeRS-Hyperactivity/Impulsivity symptom scores (that differed slightly between groups) as covariates did not affect the three-way interaction effect: (F(1,85) = 6.0, *p* = .016, η_p_^2^ = .07) and (F(1,85) = 7.7, *p* = .007, η_p_^2^ = .08), respectively. The three-way interaction also stayed significant after including the OSPAN score as a covariate (F(1,82) = 5.1, *p* = .03, η_p_^2^ = .06); of three participants no OSPAN scores were available.

#### 3.3.3. Effects on task performance

Attention Group (2) x Mood Induction (2) analyses were performed for SART task performance (target accuracy and reaction time and non-target (digit 3) commission errors). Task data of one participant in the ADHD-IA symptom–negative mood induction group was disregarded due to using wrong response buttons. None of the performance measures showed any significant main or interaction effects (*p*-values between .11 and .88).

#### 3.3.4. Correlations mood-mind wandering effects and rumination scores

To test whether the increase in TUT’s in the SART after negative mood induction in the ADHD-IA symptom group related to their earlier reported higher intrusive rumination response style, correlations between ERRI-intrusive (and deliberate) rumination scores and TUTs were computed. There was one outlier in the low symptom group on the ERRI-intrusive scale with a score of 21 (greater than 3 times the Inter Quartile Range), that was excluded from the correlation analysis. After negative mood induction, TUT’s correlated with ERRI-intrusive (but not deliberate, *p*>.50) rumination scores in the ADHD-IA symptom group (r(23) = .42, *p* = .047), but not in the low symptom group (*p*’s > .52).

### 3.4. Reading task

#### 3.4.1. Effects on mind wandering measured by online thought probes

For means (and SDs) of all reading task variables in both groups and mood induction conditions see Table 2. A 2 (Attention Group) x 2 (Mood Induction) x 2 (Time-on-Task: computed as off-task thoughts reported over the first 12 versus the last 13 thoughts probes) mixed repeated measures ANOVA was performed on mind wandering frequency as measured by the *online thought probes* in the reading task. The Time-on-Task factor was included based on multiple earlier reports of increases in mind wandering frequency over time in longer duration tasks or lectures ([52–55]. The ANOVA analysis yielded a main effect of Time-on-Task (*F*(1,86) = 27.8 *p* < .001, η_p_^2^ = .24) which was qualified by a Time-on-Task x Attention Group interaction effect (*F*(1, 86) = 7.3, *p* = .008, η_p_^2^ = .08). Follow-up tests showed that in the first half of the reading task, significantly more mind wandering was reported by participants in the ADHD-IA symptom group than by those in the low symptom group (*t*(88) = 2.3, *p* = .02), whereas mind wandering frequency was equal between the groups in the second half of the reading task (*t*(88) = 0.28, *p* = .78). This was due to a strong increase in MW frequency from the first to the second half of the task in the low symptom group (t(43) = -5.0, *p* < .001), whereas in the ADHD-IA symptom group the increase in MW with time on task was significant, but less steep due to already higher scores in the first half of the task (t(45) = -2.1, *p* = .042). No main or interaction effects of Mood Induction were found (all *p*-values ≥ .21). Inclusion of BDI, ACTeRS-Hyperactivity/Impulsivity scores and OSPAN working memory scores as a covariates did not change any of the results; Time-on-Task x Attention Group interaction remained significant: F(1,85) = 6.5, *p* = .013, η_p_^2^ = .07, F(1,85) = 6.7, *p* = .011, η_p_^2^ = .07 and F(1,82) = 7.1, *p* = .009, η_p_^2^ = .08, respectively).

#### 3.4.2. Effects on mind wandering measured by retrospective report (DSSQ)

To measure Attention and Mood effects on *retrospective reports of TRI’s and TUT’s* during the reading task, a 2 (Attention Group) x 2 (Mood Induction) x 2 (Thought Content: TRI vs TUT) mixed ANOVA was performed. This analysis yielded a main effect of Thought Content (*F*(1,86) = 119.1, *p* < .001, η_p_^2^ = .58), indicating higher reports of TRIs than TUTs. There also was a main effect of Attention Group (*F*(1,86) = 5.2, *p* = .03, η_p_^2^ = .06), indicating higher TRI and TUT during task performance in the ADHD-IA symptom group. Whereas the interaction effect between Attention Group x Thought Content was not significant (*p* = .40), post-hoc testing of Attention Group effects split up for TRI and TUT’s showed that the groups only differed significantly in reported TUT’s (t(88) = 2.8, *p* = .006) and not TRI’s (t(88) = 1.2, *p* = .24). There were no other main or interaction effects (all *p*’s > .37). Removal of one outlier in the low symptom group with an extreme score of 27 (greater than 3 times the Inter Quartile Range) on the DSSQ-TUT yielded the same ANOVA outcome: the main effect of Attention Group only became stronger (F(1,85) = 6.9, *p* = .01, η_p_^2^ = .075); Attention Group difference in TUT’s (t(87) = 3.8, *p* < .001). Inclusion of BDI, ACTeRS Hyperactivity/Impulsivity score and OSPAN scores did not change above results and the main effect of Attention Group remained significant (*p* = .03, .02 and .02 respectively).

#### 3.4.3. Effects on task performance

Attention group and Mood induction effects on *text comprehension* were studied by performing a 2 x 2 univariate ANOVA on scores obtained on the questions. A main effect of Attention group (F(1,86) = 4.8, *p* = .031, η_p_^2^ = .05) indicated lower comprehension scores in the high ADHD-IA symptom group than in the low symptom group (see Table 2). There was a tendency for this difference to be larger after negative mood induction, but the Attention group x Mood Induction effect: F(1,86) = 3.6, *p* = .06, η_p_^2^ = .04) was only marginally significant. None of the other main or interaction effects reached statistical significance.

Importantly, text comprehension scores were moderately negatively correlated with mind wandering frequency as measured by thought probes: r(90) = -.25, *p* = .02; when looking at time on task effects (because the groups only differed in mind wandering in the first half of the reading task), only mind wandering in the first half of the task appeared to be negatively correlated with reading comprehension scores r(90) = -.35, *p* = .001 (second half of the task: r(90) = -.14, *p* = .19). Furthermore, in the reading task mind wandering scores obtained via thought probes and via the DSSQ (the one outlier excluded) were moderately positively correlated: r(89) = .22, *p* = .03.

#### 3.4.4. Correlations between mind wandering effects and rumination scores

To explore possible relationships between enhanced TRI’s and TUT’s during reading performance in the ADHD-IA symptom group and type of rumination style, correlations were performed between total-DSSQ (because overall ANOVA showed significant group differences in both TUT and TRI) and ERRI rumination scores in the two attention groups. In the high ADHD-IA symptom group total DSSQ scores correlated positively with both ERRI-Intrusive and ERRI-Deliberate rumination scores, respectively r(46) = .31, *p* = .04 and r(46) = .33, *p* = .03. In the low symptom group there were no correlations between total DSSQ scores and ERRI-rumination scores (lowest *p* > .30), inclusion of the DSSQ outlier (with intrusive score of 27) in this group did not change results. In neither group were there significant correlations between reading task thought probe mind wandering scores and rumination scores (*p* > .16)

## 4. Discussion

The present study investigated the effects of mood induction on mind wandering during cognitive task performance in non-diagnosed college students with high or low ADHD-Inattention (ADHD-IA) symptoms (with ADHD-Hyperactivity/Impulsivity scores in the normal range). Also possible influences of ruminative thinking style and working memory capacity were investigated.

First, analyses of the questionnaire data showed significantly higher self-reported frequencies of daydreaming (DDFS), daily attention errors due to mind wandering (ARCES) and lower levels of trait mindfulness (MAAS) in the high ADHD-IA symptom group, compared to the low ADHD-IA symptom group. These findings replicate earlier reports of positive relations between ADHD-symptomatology and trait mind wandering levels in non-clinical student samples [11–13]. The present results however add to this literature by (to our knowledge) providing first evidence for a specific relation between ADHD *inattention* symptoms and mind wandering by using an experimental between-subjects design and including non-clinical groups of college students distinguished by the presence of high or low ADHD-Inattention symptoms, while Hyperactivity-Impulsivity symptoms were in the normal range and were matched/controlled for between groups. Also higher depression and intrusive rumination symptoms were found in the ADHD-IA symptom group, suggesting co-occurrence of ADHD-IA symptoms with higher levels of dysphoric mood and maladaptive ruminative response styles in a non-clinical student sample.

Besides group differences in self-reported trait levels of daydreaming/mind wandering as measured by questionnaires, the ADHD-IA high symptom group also showed higher mind wandering during performance of the sustained attention (SART) and reading tasks, in the SART only after negative mood induction. In both cognitive tasks, mind wandering was measured by online presented thought probes and by an often used post-task questionnaire assessing the frequency of task-related interfering (TRI) and task-unrelated thoughts (TUT’s). In the mind wandering literature, especially TUT’s (e.g. thoughts about one’s family, future or past events, or about personal worries or emotional events) are considered as (often undeliberate) instances of mind wandering. Task related interfering thoughts (TRI), referring to thoughts/worries about one’s current task performance, are on the other hand usually not regarded as instances of spontaneous mind wandering due to their task-related content, [6], even though they are often also detrimental for task performance.

A significant three-way interaction effect showed that in the SART, participants in the high ADHD-IA symptom group reported a higher frequency of TUT’s (but not TRI) on the post-task questionnaire after negative, compared to positive, mood induction while mood induction had no effects in the low symptom group. These results replicate findings from a mood induction study in healthy students without inattention symptoms, also reporting an increase in TUT’s (but in this study also TRI’s were enhanced) as measured by the same retrospective mind wandering measure after negative, but not positive, mood induction ([20, 21]: experiment 1). The current study did however not replicate the finding of negative mood specifically enhancing past-related TUT’s, as opposed to present or future related TUT’s, as measured by online thought probes in the SART ([21]: experiment 2). No effects of mood induction or attention group were found on the temporally oriented (present, future, past) online thought probes in the current SART. It should however be noted that the negative mood induced increase in past-related TUT’s reported on thought probes in the second experiment in the Smallwood and O ‘Connor [21]study, was only seen in those with relatively higher BDI scores, whereas in the current study BDI scores did not moderate thought probe (or DSSQ) results. These combined results suggest that the retrospective mind wandering measure might be more sensitive to detect effects of negative mood (induction) on mind wandering frequency (also in non-depressed people), perhaps due to its measurement of more diverse types of TUT’s. The TUT-scale of the DSSQ-retrospective mind wandering questionnaire consists of 8 items related to self, other, past and future thoughts and also includes negative-mood related items referring to the frequency of personal worries and feelings of guilt or anger. Such a conclusion would fit with reports that, besides a temporal orientation towards the past, also the social content of TUT’s, such as TUT’s involving others instead of self, is an important predictor of a negative mood state [56]. Future mood/rumination–mind wandering studies should also include emotion or mood -related thought probe categories.

So, on the basis of the present results it can be concluded that a negative mood state specifically enhanced off-task thoughts during a low resource demanding sustained attention task in students with high ADHD-IA symptoms. Such effects were found after controlling for hyperactivity/impulsivity symptoms and thus, in this student population, seem to be specifically related to inattention symptoms. Another important finding was that individual differences in working memory capacity did also not moderate the above results in any way. Although the emotional valence or intrusiveness of TUT’s was not directly measured in the current study, these results make it tempting to speculate that the current negative-mood induced increase in TUTs in the inattentive group was for a large part caused by enhanced intrusive negative thoughts (intrusive rumination or brooding). Accordingly, correlation analyses showed that only in the ADHD-IA symptom group self-reported intrusive trait rumination scores were positively correlated with the frequency of TUT’s (and not TRI) in the SART after negative mood induction. Other studies also provide support for such a conclusion by reporting difficulties with suppressing intrusive unwanted thoughts only after negative (vs positive) mood induction [57] or showing (by experience sampling) that the emotional content of mind wandering was predicted by the emotional valence of one’s preceding mood state [58]. Although these results have to be replicated in future research, these findings of negative-mood induced enhanced TUT’s in students with high ADHD-IA symptoms and an enhanced ruminative response style, provide potentially important information for educational and clinical practice. They namely suggest that some of the attention/academic problems shown by a nonclinical sample of college students with high levels of self-reported ADHD inattention symptoms, might be due to maladaptive ways to deal with negative affect (such as when having an intrusive ruminative response style), rather than to working memory/cognitive capacity problems. Consequently, in such cases other ways of remediation like mindfulness training or cognitive behavioral therapy might be needed, that have been shown to be successful in reducing mind wandering or negative thinking in healthy adults [24, 59]. Further studies are needed to investigate whether current findings generalize to individuals meeting diagnostic criteria for ADHD.

In the reading task more frequent mind wandering in the ADHD-IA, compared to the low symptom group, was reported on both the online thought probes and the post-task questionnaire, the latter now indicating an increase in both TRI and TUT. No effects of mood induction on mind wandering during reading were found, but, comparable to the SART findings, a ruminative response style was positively correlated with mind wandering frequency as measured by the post-task questionnaire only in the ADHD-IA symptom group. However, now both deliberate and intrusive rumination scores were positively correlated with both TRI and TUTs. These findings might suggest that the higher self–reported tendency to ruminate in the ADHD-IA symptom group could have led to a more general increase in self-generated thoughts about personal concerns/worries about their task performance (TRI) as well as about personal task-unrelated issues (TUT). The fact that, as opposed to in the SART, also interfering thoughts about task performance were enhanced might have to do with the higher complexity of the reading task, perhaps rising more “fear-of-failure” thoughts in the ADHD-IA symptom group. Such an explanation matches the significantly worse text comprehension scores shown by those with high ADHD-IA symptoms, indicating that the task was indeed harder for them. An earlier correlational study [12] that did not include extreme ADHD symptom groups did not find a correlation between ADHD symptoms and text comprehension scores.

The online thought probe analyses in the reading task, now only asking yes/no responses to the question if one had task-unrelated thoughts, revealed a three way interaction effect, confirming higher mind wandering in the ADHD-IA symptom group than the no-symptom group especially during the first half (about 16 minutes) of the task. In the second half of the reading task mind wandering stayed at the same level in the ADHD-IA group but increased in the low symptom group, abolishing group differences in the last part of the task. Such a rise in mind wandering frequency across time in longer duration tasks in healthy young adults has been frequently reported before in reading and other tasks [26, 52–55]. The deviancy in the ADHD-IA high symptom group thus lies in the fact that they started mind wandering right from the start of the reading task. Consistent with prior results in healthy adults [28, 29, 51, 60, 61], we also found a moderate negative correlation (r = -.25) between mind wandering frequency (measured by thought probes) and text comprehension scores, which suggests that higher levels of mind wandering were at least partially responsible for worse text comprehension. Previous studies have suggested that mind wandering negatively affects text comprehension by interrupting or disturbing the formation of a situation model of the text [62, 63]. Such an explanation would fit the present findings since the earlier one starts to wander off during reading, the higher detrimental effects on subsequent situational model building and text comprehension are expected. Smallwood et al., [51] indeed reported that mind wandering during early parts of the story had more detrimental effects on text comprehension. This was confirmed by our data, showing that only mind wandering scores in the first half of the task, where group differences in mind wandering were found, correlated negatively (r = -.35) with text comprehension. Finally, no correlations between thought probe scores and rumination scores were found. It should be noted that the negative mood induced rise in mind wandering in the ADHD-IA symptom group did not lead to worse task performance in the SART as opposed to its detrimental effects on text comprehension in the reading task. This absence of mind wandering consequences on SART performance is however in compliance with a recent meta-analysis study [64], only showing negative relationships between mind wandering and attention performance on more complex and longer duration (> 30 minutes) tasks than the SART, because only the former tasks need all available attentional resources to maintain adequate performance levels over time ([65].

It is not clear why effects of negative mood on mind wandering in the ADHD-IA symptom group were only found in the SART and not the reading task. It is unlikely that this is due to a lack of effectivity of our mood induction procedure; highly significant decreases in positive mood and increases in negative mood were found after the first negative mood induction. However, whereas additional mood measurements showed that this negative mood induction effect sustained until after the SART, it somewhat decreased after the reading task, while at the same time negative mood increased over time in the positive mood induction group, reducing group differences in mood state after the reading task. The reading task might have been too long for mood induction effects to sustain (it was about three times longer than the SART), although longer term effects of negative mood inducing procedures have been reported in depression-vulnerable persons [66, 67]. This was however, to the best of our knowledge, the first mood-induction–mind wandering study including a more complex reading task besides the SART. Another explanation, although purely speculative at this stage and needing further research, might be that the focus on following a story in the reading task on which one would be later questioned, might have prevented/distracted participants from negative mood-induced, intrusive mind wandering.

A last important point to note is that, despite multiple earlier reports of links between working memory capacity and ADHD-inattentiveness and/or mind wandering, the current attention groups did not differ in working memory capacity as measured with the operation span task and mean scores in both groups also fell within the normal population range (see Redick et al., 2012). Accordingly, covariance analyses including working memory capacity did not affect the above reported group differences in mind wandering or mood induction effects. Whereas prior ADHD–mind wandering studies did not consider the role of working memory capacity, Franklin et al. [12] reported zero correlations between reading span and ADHD-symptom scores in their student sample. Furthermore, another study [68] reported developmental differences in working memory capacity between adolescents and adults that were not accompanied by differences in mind wandering frequency during the SART. On the basis of the current results it can be concluded that the increased mind wandering in the present non-clinical sample of college students with high ADHD-inattention symptoms is not due to a working memory/attentional control deficit perse. It rather seems due to a problem with the maintenance and reorientation of attentional resources on task-relevant information in the presence of distracting, task-unrelated thoughts induced by negative emotionality. This would be in line with prior electrophysiological evidence for a role of differences in attention allocation strategies rather than a shortage of capacity underlying deficient divided attention performance in children with ADHD [69].

### Conclusion and limitations

The present study to our knowledge provides first initial/preliminary evidence for negative-mood induced increases in mind wandering during sustained attention performance in students with high ADHD-Inattention symptoms. These results will however need replication considering that mood effects were only found in the SART and not the reading task and on only one of the two included mind wandering measures. It should also be investigated whether these results generalize to individuals meeting diagnostic ADHD criteria. Furthermore, whereas mood effects were based on experimental manipulations, current conclusions regarding the role of ruminative response styles in the ADHD-mood-mind wandering relations are based on correlation analyses that, regarding the relatively small samples sizes, need replication in larger samples. Finally, in future studies, next to ruminative response styles, also the ruminative nature of responses *during* task performance should be measured/manipulated since higher trait rumination scores may not always predict higher state rumination responses during negative affective or stressful situations [70, 71].
